# National Association of Dental Examiners

**Published:** 1885-09

**Authors:** 


### NATIONAL ASSOCIATION OF DENTAL EXAMINERS.

The National Association of Dental Examiners held its fourth session in Curtiss Hall, Minneapolis, Minn., commencing Tuesday, August 4, 1885. President J. Taft in the chair.

The following State boards were represented, the four last named being new members: Ohio, by J. Taft and H. A. Smith; Illinois, by Geo. H. Cushing, A. W. Harlan, and C. A. Kitchen; Pennsylvania, by E. T. Darby; Maryland, by T. S. Waters; Michigan, by G. R. Thomas and A. T. Metcalf; Louisiana, by Joseph Bauer; Indiana, by S. B. Brown; Iowa, by W. P. Dickinson, J. T. Abbott, J. Hardman, J. F. Sanborn, and E. E. Hughes; Dakota, by S. J. Hill; Kansas, by L. C. Wasson and Wm. Shirley; Wisconsin, by Edgar Palmer, C. C. Chittenden, B. G. Marcklein, E. C. French, and J. S. Reynolds; Minnesota, by S. T. Clements and G. V. I. Brown.

The following boards belonging to the association were not present: Vermont, New Jersey, Georgia, West Virginia, Mississippi, South Carolina and Kentucky.

The following resolutions were adopted:

*Resolved*, That this association most earnestly commends the action of the Wisconsin and other State Boards of Dental Examiners, in refusing to accept the diplomas of the so-called Wisconsin Dental College located at Delavan, on the ground that it is not a reputable school, and recommends to all State boards to which the diplomas of that institution shall be offered that they likewise refuse them.

*Resolved*, As the sense of this association, that persons engaged in the study of dentistry and physicians practicing as such should not be considered eligible to registration as dentists.

*Resolved*, That this association recommends that all applicants holding diplomas from the Royal College of Dental Surgeons of Ontario be required to submit to examination before they are granted license to practice.

WHEREAS, The dental law of the State of Maryland seems to be restrictive in its character; it is the sense of this body that the dental profession of said State of Maryland should, at the next session of its Legislature, seek to cause said dental law be so amended as to be in harmony with the dental laws of the other States.

*Resolved*, That the secretary be instructed to forward a copy of the above resolution to the State Board of Dental Examiners of Maryland.

*Resolved*, That this association recommend all State Boards not to grant temporary licenses to first-course students, or any others, unless fully satisfied that such applicants have had at least two years of practical clinical instruction. Such applicants shall pass as well a proper theoretical examination.

The following officers were then elected for the ensuing year: J. Taft, president; T. S. Waters, vice-president; George H. Cushing, secretary and treasurer.

Adjourned to meet at the place to be selected for the next meeting of the American Dental Association, on the Monday preceding the meeting of that body.

